# Celecoxib Sensitizes *Staphylococcus aureus* to Antibiotics in Macrophages by Modulating SIRT1

**DOI:** 10.1371/journal.pone.0099285

**Published:** 2014-06-20

**Authors:** Madhavi Annamanedi, Arunasree M. Kalle

**Affiliations:** Department of Animal Sciences, School of Life Sciences, University of Hyderabad, Hyderabad, India; Boston University, United States of America

## Abstract

We have previously shown that celecoxib in combination with an antibiotic, increase the bacterial sensitivity to antibiotics. However, the underlying molecular mechanism remained elusive. Efficacy of the combinatorial treatment of celecoxib and ampicillin *in vitro* was evaluated on macrophage-phagocytosed *S aureus*. To elucidate the mechanism, signaling pathway of infection and inflammation involving TLR2, JNK, SIRT1 and NF-κB was studied by FACS, Western blot, ELISA and activity assays. Combinatorial treatment of ampicillin and celecoxib reduced the bacterial load in the macrophages. Further studies clearly suggested the activation of the master regulator of oxidative stress and inflammation SIRT1,, by celecoxib when used alone and/or in combination with ampicillin. Also, the results indicated that celecoxib inhibited JNK phosphorylation thereby stabilizing and activating SIRT1 protein that inhibited the COX-2 gene transcription with a significant decrease in the levels of protein inflammatory cytokines like IL-6, MIP-1α and IL-1β *via* inhibition of NF-κB. SIRT1 activation by celecoxib also resulted in increase of catalase and peroxidase activity with a decrease in Nitric oxide levels. In conclusion, we demonstrate a novel role of celecoxib in controlling inflammation as an enhancer of antibiotic activity against bacteria by modulating SIRT1.

## Introduction

Microbial drug resistance is of increasing concern and in the light of time and cost incurred in the novel drug development; the current research is focused on identifying alternate therapies by drug repurposing and repositioning. We have shown that celecoxib, a cyclooxygenase-2 (COX-2)-specific inhibitor, in combination with an antibiotic increased bacterial sensitivity to the antibiotic [1]. Recently it has been demonstrated that a combination therapy using antibiotics and already approved drugs can overcome bacterial resistance [2]. However, *in vitro* effect of such combination therapy on phagocytosed bacteria and the underlying molecular mechanism is not yet demonstrated.

Recently, role of human SIRT1 in inflammation and as oxidative stress regulator has been demonstrated [3]–[7]. SIRT1 regulates proinflammatory gene expression including COX-2, TNF-α, interleukins etc. Furthermore, direct association of SIRT1 with COX-2 gene promoter has been observed recently suggesting that SIRT1 as an anti-inflammatory regulatory protein [8]. It was also demonstrated that SIRT1 is down regulated and COX-2 is over expressed in macrophages infected with bacteria [3], [8].

With this background the present study was done to evaluate the possibility of limiting bacterial survival in macrophages using a combination of COX-2 inhibitor, celecoxib and an antibiotic, ampicillin, *via* SIRT1. The results showed that combination treatment reduced bacterial survival in macrophages and that celecoxib modulated SIRT1 activity. Combinatorial treatment reduced TLR2 activation by *S aureus* and also inhibition of JNK and NF-κB. The proinflammatory cytokines like PGE_2_, IL-6, IL-2 and IL-1β levels decreased significantly in the presence of celecoxib and increased in the presence of suramin, a SIRT1 inhibitor thus confirming celecoxib effect *via* SIRT1 modulation. Increased SIRT1 activity also increased the activity of oxidative stress enzymes such as catalase and peroxidase and decreased the levels of Nitric oxide and thus infection.

## Materials and Methods

### Bacterial strains, cells and cell culture

Gram-positive bacteria, *Staphylococcus aureus* (SA; ATCC-29213) were grown in Brain-Heart Infusion medium. Mouse macrophages, RAW 264.7, cells were obtained from NCCS, Pune and cultured in RPMI1640 medium supplemented with 10% FBS. The cells were incubated in 5% CO_2_ at 37°C.

### Phagocytosis and intracellular bacterial viability assay

Phagocytosis of SA by RAW 264.7 was carried out as described previously [9]. After growing the cells in gentamycin for 24 h to eliminate extracellular bacteria, the cells were then incubated with or without celecoxib (10 µM), ampicillin (5 µg/ml) or both for 12 h in RPMI medium without antibiotics. After incubation, the cells were lysed in ice-cold distilled water and the intracellular bacterial viability was assessed using 100-fold serially diluted cell lysates by counting the number of the colony forming units (CFU) on LB agar plates.

### FACS analysis

Activation of TLR2 was studied by FACS analysis using FITC-labelled-TLR2 antibody (eBioscience, USA) as described previously [10].

### Western Blot Analysis

Immunoblot analysis and RT-PCR analysis were carried out as described earlier [11].

### Immunoprecipitation and SIRT1 activity assay

Immunoprecipitation of SIRT1 protein from whole cell lysates of RAW 264.7 cells phagocytosed with *S aureus* and treated with celecoxib, ampicillin, combination, suramin and Resveratrol using anti-SIRT1 antibody and activity assay using fluorimetric activity assay kit (Enzo Life Sciences, USA) was carried out as described earlier [12].

### Measurement of p65 levels of NF-κB

Activation of NF-κB was quantified by the measuring the levels of p65 subunit in the cell lysates using NF-κB p65 measuring kit from Cell Signaling Technologies, USA as per manufacturer's protocol. The p65 levels are measured in cytoplasm and nuclear isolates of *S aureus*-phagocytosed RAW 264.7 cells treated with or without drugs.

### SIRT1 Activity Assay

SIRT1 activity assay was carried out using Fluor-de-lys SIRT1 activity assay kit from Enzo Life Sciences as per manufacturer's protocol.

### NO, Peroxidase and Catalase assay

Catalase and peroxidase activity in the whole cell lysates was determined as previously described [13]. NO levels were determined in the culture supernatants by Griess reagent as earlier [14].

### Cytokine analysis

Release of cytokines like MIP-1α, IL-2, IL-6, IL-1β and PGE_2_ into culture supernatants was analyzed using Quantikine immunoassays kits (R&D Systems, USA) according to manufacturer's instructions.

### In silico Analysis

As SIRT1 protein structure is unavailable, homology modeling of catalytic domain (217–415) of SIRT1 protein was done using PRIME SUITE, Schrödinger software with SIRT2 (PDB ID: 1J8F) as the template. The modeled protein was then used for docking analysis with celecoxib using Autodock 3.0 software. A docking energy score of −6.5 kCal/mol was obtained for the best pose.

### Statistical analysis

Statistical analysis was carried out using OneWay ANNOVA of the Sigmaplot software for values obtained from three independent experiments with duplicates in each and the p-value<0.01 is considered significant.

## Results

### Combination of celecoxib and ampicillin limit S aureus survival in RAW264.7 cells

**Figure 1:**
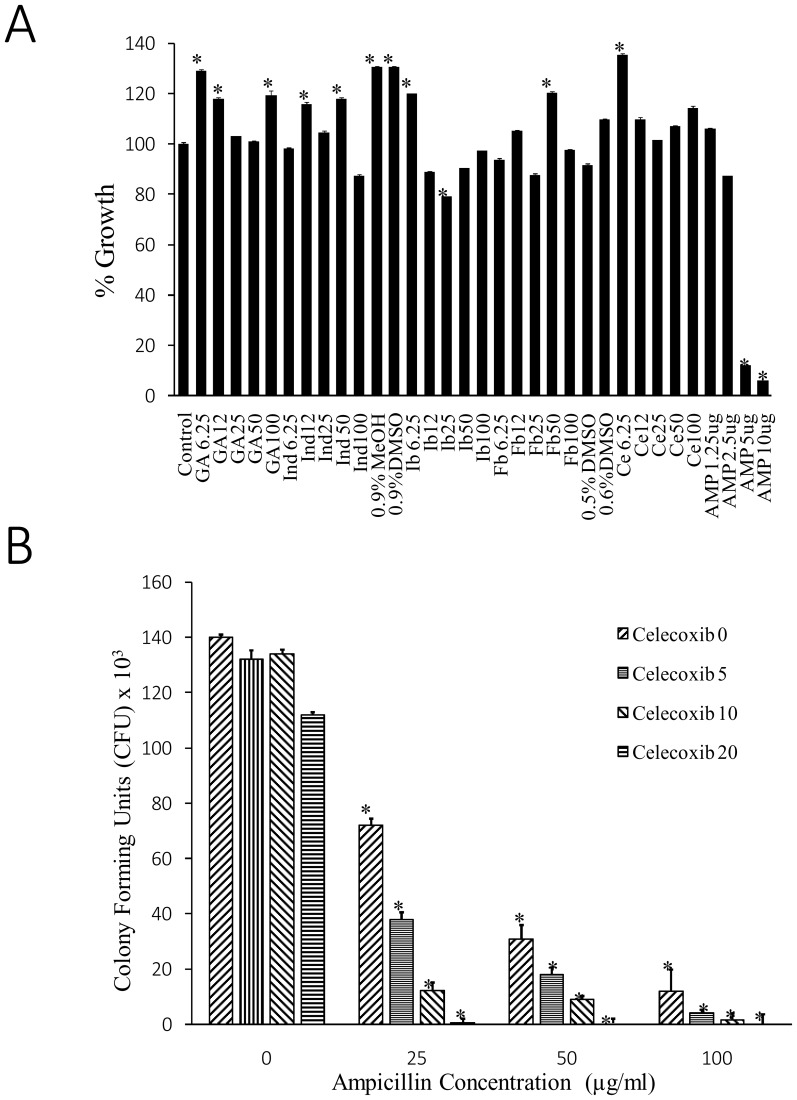
Celecoxib inhibits intracellular bacteria. A. Effect of NSAIDs such as Indomethacin (Ind), Ibuprofen (Ib), Flurbiprofen (Fb), gallic acid (GA) and celecoxib (Ce) and antibiotic ampicillin (Amp) on the growth of *Staphylococcus aureus*. DMSO and MeOH (methanol) are used as solvent controls. * p-value<0.01 compared to Control. B. Effect of celecoxib, ampicillin and their combination on the survival of intracellular bacteria as measured by the CFU. * p-value<0.01 compared to corresponding Control.

To demonstrate the *in vitro* efficacy of combinatorial treatment of ampicillin and celecoxib on phagocytosed *S aureus*, we first evaluated the effect of Non-steroidal Anti-Inflammatory Drugs (NSAIDs) alone on the growth of *S aureus* bacteria. The results indicated no inhibitory effect of NSAIDs on the growth of bacteria (Fig. 1A). Next, the combinatorial effect of celecoxib and antibiotic (ampicillin) on the macrophage-phagocytosed bacteria was tested. The results clearly demonstrated decreased bacterial survival load in the combination treatment dose dependently as indicated by the reduced colony forming units (Fig. 1B).

### SIRT1 activation by celecoxib inhibits bacterial inflammation and infection

**Figure 2:**
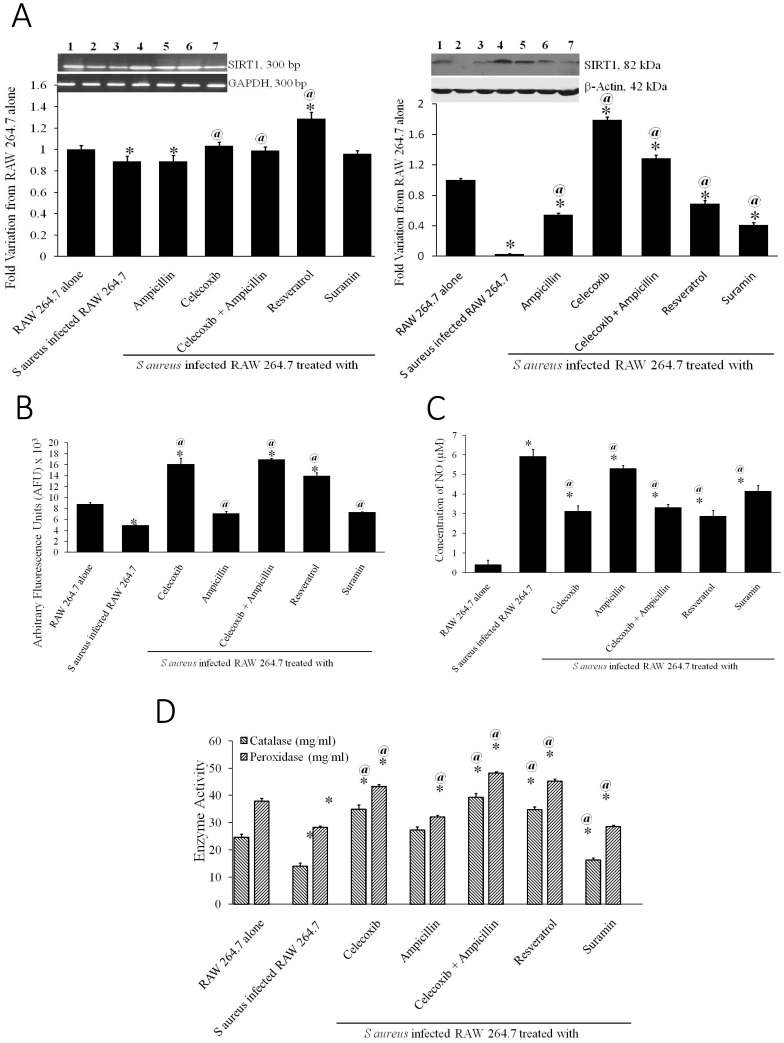
Combinatorial treatment with celecoxib activated antioxidant enzymes *via* SIRT1. A. RT-PCR and Western blot analysis of SIRT1 in RAW 264.7 cells (Lane 1) and *S aureus* phagocytosed cells (Lane 2) treated with ampicillin 100 µg/ml (Lane 3), celecoxib 10 µM (Lane 4), and combination of celecoxib and ampicillin (Lane 5), Resveratrol 50 µM (Lane 6) and Suramin 25 µM (Lane 7). GAPDH and β-actin served as controls for RT-PCR and Western blot respectively. B. Activity assay of SIRT1 immunoprecipitated from whole cell lysates of cells infected without or with *S aureus* and treated with ampicillin, celecoxib, ampicillin+celecoxib, Resveratrol and Suramin. * p-value<0.01 compared to RAW 264.7 alone, @ p-value<0.01 compared to S Aureus infected RAW 264.7. C. Nitric Oxide levels in the culture supernatants of cells infected without or with *S aureus* and treated with ampicillin, celecoxib, ampicillin+celecoxib, Resveratrol and Suramin. * p-value<0.01 compared to RAW 264.7 alone, @ p-value<0.01 compared to S Aureus infected RAW 264.7. D. Anti-oxidant enzymes, catalase and peroxidase, activity in the whole cell lysates of cells infected without or with *S aureus* and treated with ampicillin, celecoxib, ampicillin+celecoxib, Resveratrol and Suramin. * p-value<0.01 compared to RAW 264.7 alone, @ p-value<0.01 compared to S Aureus infected RAW 264.7.

In an attempt to identify the underlying molecular mechanisms of combinatorial effect of celecoxib and ampicillin on survival of intracellular *S aureus*, we assessed the role of SIRT1, an anti-inflammatory, oxidative stress regulatory protein. SIRT1 expression at RNA and protein levels was evaluated and the results clearly demonstrated an increase in SIRT1 levels when treated with celecoxib alone or in combination (Fig. 2A). The activity assay using fluorescence-labeled substrate demonstrated a significant increase in the SIRT1 activity in presence of celecoxib (Fig. 2B). Since SIRT1 is known to inhibit Nitric oxide (NO) and activates catalase and peroxidase enzymes during oxidative stress and inflammation, we also assessed the levels of NO, catalase and peroxidase. The results clearly demonstrated a significant decrease in NO levels (Fig. 2C) and increase in antioxidant enzyme activity of catalase and peroxidase (Fig. 2D).

### Celecoxib-activated SIRT1 attenuates S.aureus infection via TLR2-JNK–NF-κB pathway

**Figure 3:**
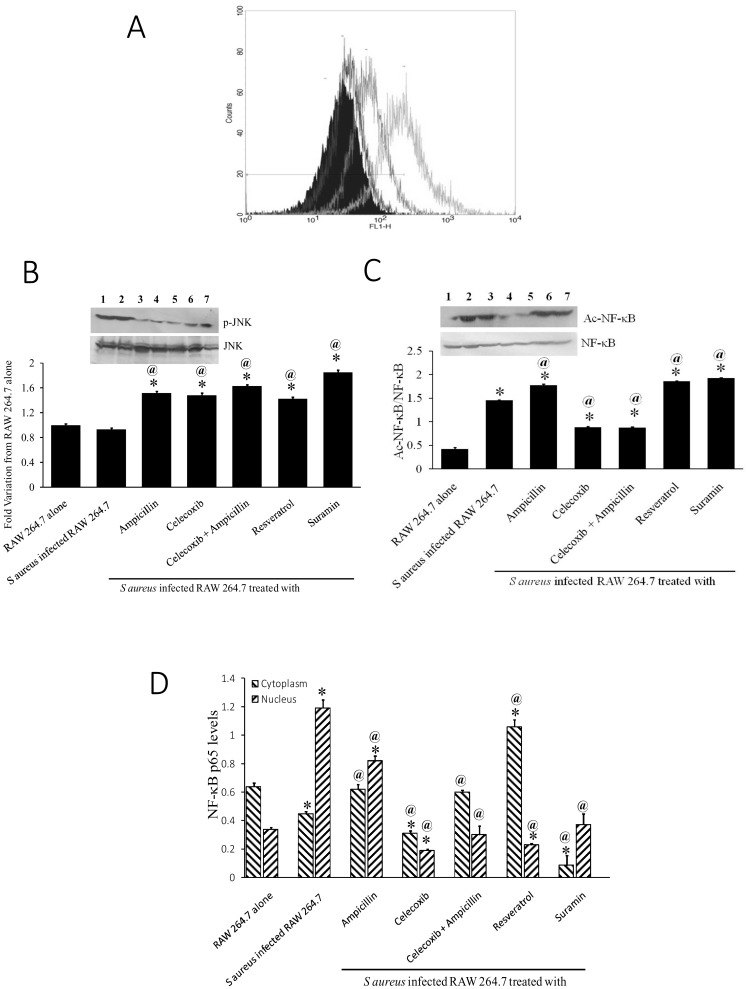
Celecoxib-induced SIRT1 activity inhibited TLR2-JNK-NF-κB signaling. A. Flowcytometer analysis of TLR2 expression in RAW-264.7 cells phagocytosed with *S aureus* (1) and treated with ampicillin (2) or celecoxib (3) or both in combination (4). B. Western blot of levels of p-JNK and JNK in *S aureus* phagocytosed cells (Lane 1) and treated with ampicillin 100 µg/ml (Lane 2), celecoxib 10 µM (Lane 3), and combination of celecoxib and ampicillin (Lane 4), Resveratrol 50 µM (Lane 5) Suramin 25 µM (Lane 6) and RAW-264.7 cells (Lane 7). C. Western blot of levels of Ac- NF-κB and NF-κB in cell lysates of cells without (Lane 1) and with phagocytosed bacteria (Lane 2) treated with ampicillin (Lane 3) or celecoxib (Lane 4) or both (Lane 5) or Resveratrol (Lane 6) or suramin (Lane 7). D. NF-κB p65 levels in cytoplasmic and nuclear isolates of cells treated with/without, and ampicillin and celecoxib. infected without or with *S aureus* and treated with ampicillin, celecoxib, ampicillin+celecoxib, Resveratrol and Suramin. * p-value<0.01 compared to RAW 264.7 alone, @ p-value<0.01 compared to S Aureus infected RAW 264.7.

Further to evaluate the specificity of SIRT1 activation by celecoxib, effect of SIRT1 on TLR2-mediated inflammatory pathway was studied. Celecoxib-induced SIRT1 resulted in down regulation of TLR2 as evidenced by decreased TLR2 expression in cells treated with celecoxib upon flowcytometer analysis (Fig. 3A). There was a marked decrease in the phosphorylation levels of JNK (Fig. 3B). SIRT1 activation led to decreased Ac-NF-κB levels indicating decrease in the NF-κB (p65) nuclear translocation (Fig. 3C). The decrease in the nuclear translocation of NF-κB was also confirmed by the levels of p65 in nuclear isolates of cells treated with celecoxib and in combination (Fig. 3D).

### Celecoxib-activated SIRT1inhibited proinflammatory gene expression

**Figure 4:**
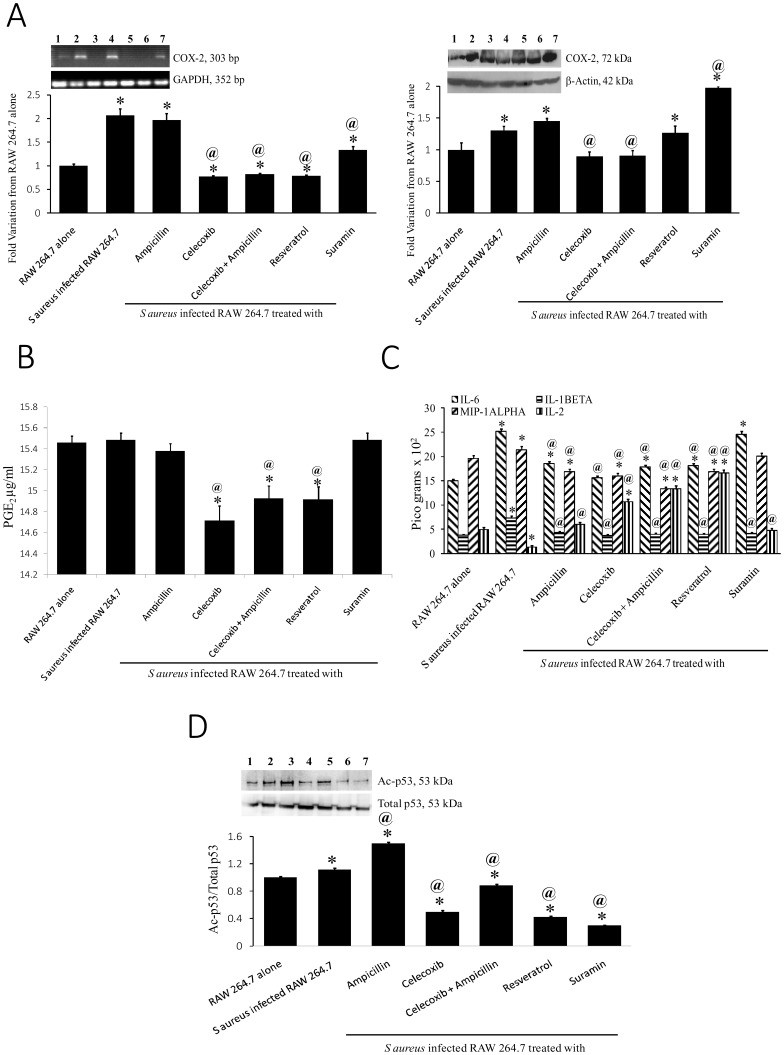
Celecoxib-induced SIRT1 activity inhibited proinflammatory gene expression. A. COX-2 RNA and protein expression in cell lysates of cells without (Lane 1) and with phagocytosed bacteria (Lane 2) treated with celecoxib (Lane 3) or ampicillin (Lane 4) or both (Lane 5) or Resveratrol (Lane 6) or suramin (Lane 7) B. PGE_2_ levels in the culture supernatants of cells infected without or with *S aureus* and treated with ampicillin, celecoxib, ampicillin+celecoxib, Resveratrol andSuramin. * p-value<0.01 compared to RAW 264.7 alone, @ p-value<0.01 compared to S Aureus infected RAW 264.7. C. Levels of IL-6, IL-1β, MIP-1α and IL-2 in the culture supernatants of cells infected without or with *S aureus* and treated with ampicillin, celecoxib, ampicillin+celecoxib, Resveratrol andSuramin. * p-value<0.01 compared to RAW 264.7 alone, @ p-value<0.01 compared to S Aureus infected RAW 264.7. D. Total p53 and Ac-p53 levels in cells treated without (Lane 1) and with phagocytosed bacteria (Lane 2) treated with ampicillin (Lane 3) or celecoxib (Lane 4) or both (Lane 5) or Resveratrol (Lane 6) or suramin (Lane 7).

Further to assess the celecoxib-induced activity of SIRT1 on inflammatory gene expression, a direct target of SIRT1, COX-2 protein and RNA levels were analyzed. The results clearly demonstrated an inverse correlation with the levels of SIRT1 indicating that activated SIRT1inhibited COX-2 gene transcription and thus protein expression (Fig. 4A). The reduction in the COX-2 protein levels also affected the activity resulting in significant decrease in the PGE_2_ levels (Fig. 4B). Furthermore, celecoxib is a known COX-2 inhibitor, thus reducing the PGE_2_ levels significantly. Activated SIRT1 also inhibited the proinflammatory cytokine expression, IL-6, IL-1β and MIP-1α. There was a significant increase in the anti-inflammatory cytokine, IL-2 levels (Fig. 4C). To further confirm the activation of SIRT1 by celecoxib, direct targets of SIRT1, p53 and Ac-p53 levels were analyzed by Western blot analyses. The blot clearly suggested activation of SIRT1 by celecoxib (Fig. 4D).

### Celecoxib activates SIRT1 directly

**Figure 5:**
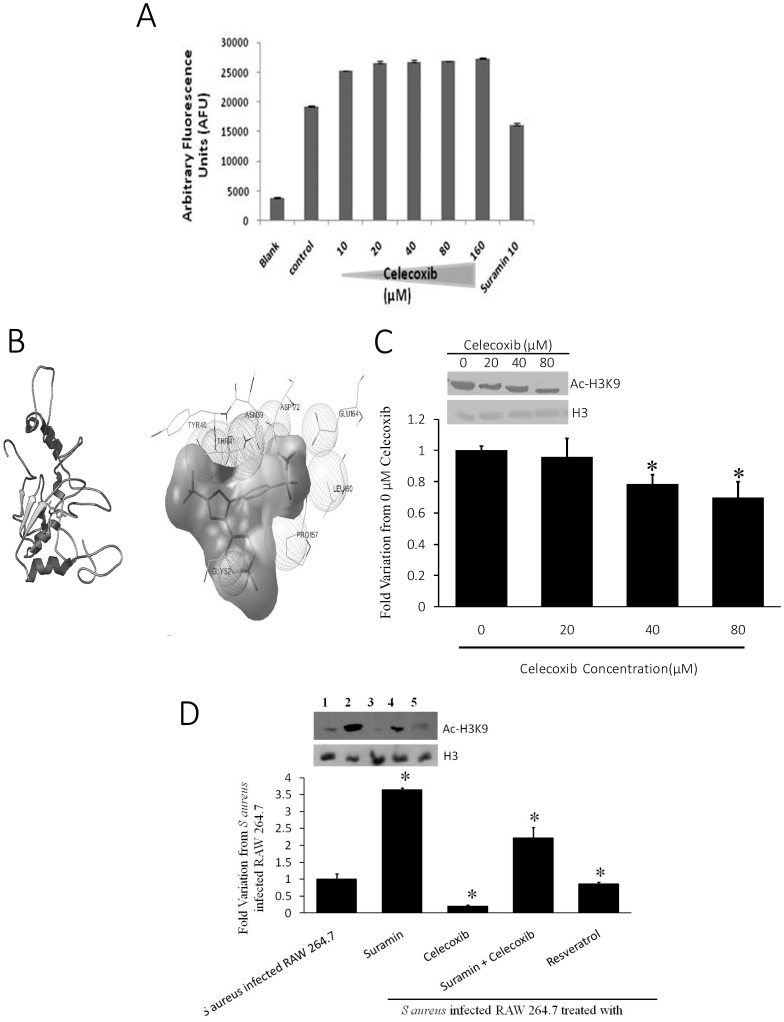
Celecoxib activates SIRT1 in a biochemical, *in silico* and *in vitro* cell-based assay. A. A representative graph of three independent experiments each with duplicates showing the enzyme activity of SIRT1 in presence and absence of different concentrations of celecoxib. Suramin, a known inhibitor of SIRT1 is taken as an assay control. * p-value<0.01 compared to Control. B. Left - Model of Celecoxib bound to SIRT1 protein (1–217 residues, proposed N-terminal activation domain of SIRT1). Right - Model showing the interactions of Celecoxib with SIRT1 protein. C. A representative Western blot showing a dose-dependent decrease of Ac-H3K9 levels in presence of celecoxib. * p-value<0.01 compared to Control. D. A representative Western blot showing the signal compensation of Ac-H3K9 levels on co-treatment of cells with celecoxib and suramin at 25 µM concentration each. * p-value<0.01 compared to Control Lane 1. 5 mM sodium butyrate treated cells. Lane 2. Suramin 25 µM. Lane 3. Celecoxib 25 µM. Lane 4. Celecoxib and Suramin 25 µM. Lane 5. Resveratrol 50 µM.

To test whether celecoxib activates SIRT1 directly or indirectly, SIRT1 activity assay was done using fluorophore-labeled Ac-p53 peptide in presence of different concentrations of celecoxib. The results demonstrated that celecoxib activated SIRT1 at 10 µM concentration. However, there was no dose dependent increase in the activation of SIRT1. (Fig. 5A). Further *in silico* docking analysis was performed using modeled SIRT1 protein (by PRIME SUITE software, Schrodinger) and celecoxib using Autodock 3.0. Autodock results showed that Celecoxib bound to the N-terminus of SIRT1with a binding score of −6.5 kCal/mol suggesting activation of SIRT1 (Fig. 5B). Next we assessed the SIRT1 activation by celecoxib in an *in vitro* cell based assay by analyzing the acetylated H3K9 levels in presence of celecoxib and suramin. The immunoblot showed a significant decrease in the levels of Ac-H3K9 in presence of celecoxib suggesting increased SIRT1 activity (Fig. 5C). This decrease was not accounted to the activation of other HDACs as class I and class II HDACs were inhibited by sodium butyrate in all the experiments. Furthermore, cells co-treated with 10 µM celecoxib and 25 µM suramin concentration showed signal compensation of Ac-H3K9 levels further confirming the activation of SIRT1 by celecoxib *in vitro* (Fig. 5D).

## Discussion

Restricted use of current antibiotics and development of new classes of antibiotics are thought to reduce emergence of drug-resistant bacteria [15]. However, the probability of resistance to the new class of antibiotics cannot be neglected. In such a scenario, evaluating the efficacy of combination therapy of an antibiotic and a FDA drug having no target in bacteria might help reduc the risk of resistance development. We have previously shown that Celecoxib, a selective COX-2 inhibitor having no target in bacteria, could increase the sensitivity of bacteria to antibiotics when given in combination to the laboratory strains of bacteria [1]. But, the efficacy and mechanism of action of such combination on intracellular bacteria and host response need to be evaluated to further clinic studies. In the present study, we made an attempt to study the underlying molecular mechanism of combination therapy of celecoxib and ampicillin on macrophage-phagocytosed *S aureus*.

Cyclooxygenase-2 (COX-2) has been identified as a key player in inflammation. Due to the gastric side effects of Non-steroidal anti-inflammatory drugs (NSAIDs) *via* inhibition of COX-1; COX-2 inhibitors and coxibs gained much importance in treating inflammatory diseases [16]. Celecoxib is one of the known COX-2 inhibitors and its anti-inflammatory effects are very well demonstrated [17], [18]. We first evaluated the effect of NSAIDs including celecoxib alone on the growth of *Staphylococcus aureus* bacteria and the results indicated no inhibitory effect on the growth of the bacteria.

Next, the combinatorial effect of celecoxib and antibiotic (ampicillin) on the macrophage-phagocytosed *S aureus* was tested. The results clearly demonstrated the decreased bacterial survival load in the combination treatment dose dependently and results were consistent with previous studies [19].

Silent information regulator 2 homolog, SIRT1, is known to play a key role in inflammaging [20] and inflammation [21]. SIRT1 has been shown to regulate NF-κB, a key transcriptional regulator of inflammation [22] *via* inhibition of JNK and ERK1/2 pathway. Recent studies have also shown down regulation of SIRT1 in inflammatory tissues including macrophages infected with bacteria [23]. Studies have shown that pharmacological activation of SIRT1 by resveratrol which helps in resolution of inflammation [3]. SIRT1 is also known to regulate COX-2 gene transcription directly [24]. Therefore, we evaluated the role of SIRT1 in the combinatorial efficacy to treat bacterial infection. The SIRT1 RNA and protein levels were significantly reduced in bacteria-phagocytosed macrophages. However, there was a significant increase in cells treated with celecoxib alone or in combination (Fig. 2A). Further study using bacteria-infected macrophage cell lysate showed a significant increase in the SIRT1 activity in presence of celecoxib and in combination (Fig. 2B) directly correlating with the RNA and protein levels. Similar results were obtained in a study using resveratrol [25].

TLR-2 is involved in recognizing *S aureus* during infection [26]. Therefore we also studied the expression of TLR2 in combination treatment in macrophages and our results, along with other studies [27], showed that celecoxib indeed inhibited TLR-2 expression. The downstream molecule affected by TLR-2 signaling is NF-κB and NF-κB is involved in regulation of many physiological and pathophysiological processes including inflammation and cancer [28], [29]. Acetylation of NF-κB by p300 histone acetyl transferase is required for its DNA-binding activity and SIRT1 is known to deacetylate NF-κB thereby inhibiting its activity [30]. Resveratrol is also shown to inhibit NF-κB activity by SIRT1 activation [31] and our results are assenting those studies.

Since SIRT1 is known to repress COX-2 gene expression, we tried to see the effect of SIRT1 activation on COX-2 gene and protein expression. Results clearly indicated a decrease in COX-2 RNA and protein expression and also activity. Our results in the present study are in agreement with the previous reports [8], [32]–[35]. Using biochemical and *in silico* assays we demonstrate that celecoxib activates SIRT1 by binding to its N-terminal domain. Previous studies have shown that natural *in vitro* activator of SIRT1, Activating regulator of SIRT1 (AROS) also binds to N-terminus of SIRT1 [36]. Furthermore, other known activators of SIRT1 (SRT1720, resveratrol) are also shown to bind to N-terminal SIRT1 [37], [38] and thus our results are also in accordance with these studies implying activation of SIRT1 by celecoxib. Previously it was shown that acetylated histone H3 at lysine 9 (H3K9) is a preferred substrate of SIRT1 [39]. Here, the *in vitro* SIRT1 activation by celecoxib was demonstrated by decrease in H3K9 acetylated levels which is in agreement with previous studies. Further this was confirmed by the cotreament studies using celecoxib and suramin which showed signal compensation in the Ac-H3K9.

## Conclusions

**Figure 6:**
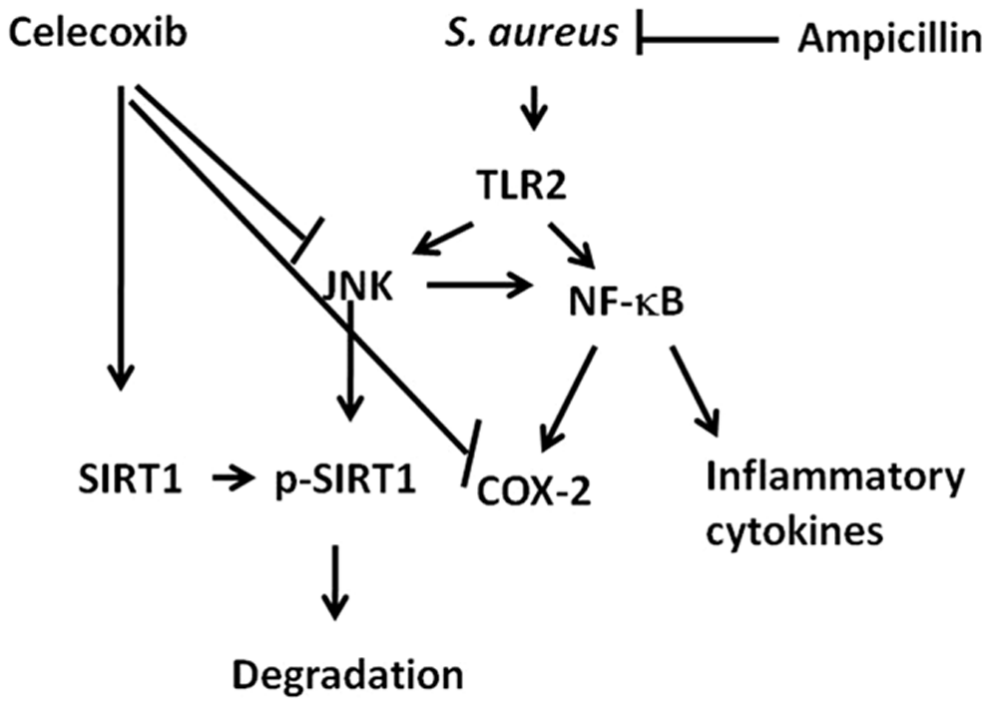
Schematic representation of mechanism of action of combinatorial treatment in host cells.

We demonstrate that celecoxib sensitizes intracellular *S aureus* to antibiotic thereby limiting bacterial survival and infection. Celecoxib activates SIRT1 in macrophages infected with *S aureus*, thus inhibiting inflammatory gene expression such as COX-2, NO, IL-6, MIP-1α, IL-1β *via* TLR2, JNK and NF-κB pathways (Fig. 6). In conclusion, the present study signifies the efficacy of the combinatorial treatment of Celecoxib and antibiotics to treat *S aureus* infection.
